# Mucoadhesive Interpolyelectrolyte Complexes for the Buccal Delivery of Clobetasol

**DOI:** 10.3390/polym10010085

**Published:** 2018-01-17

**Authors:** Venera R. Garipova, Chiara G. M. Gennari, Francesca Selmin, Francesco Cilurzo, Rouslan I. Moustafine

**Affiliations:** 1 Department of Pharmaceutical, Analytical and Toxicological Chemistry, Kazan State Medical University, 49 Butlerov Street, 420012 Kazan, Russia; venero4ka_87@mail.ru (V.R.G.); 2 Department of Pharmaceutical Science, University of Milan, Via G. Colombo 71, 20133 Milan, Italy; chiara.gennari@unimi.it (C.G.M.G.); francesca.selmin@unimi.it (F.S.); francesco.cilurzo@unimi.it (F.C.)

**Keywords:** Carbopol, clobetasol, Eudragit^®^ E PO, interpolyelectrolyte complex, mucoadhesion, oral lichen planus, oral lyophilisates, maltodextrin, resuspendibility

## Abstract

This work aimed to investigate the feasibility to design: (a) a mucoadhesive interpolyelectrolyte complex (IPEC) loaded with clobetasol propionate (CP) intended to treat oral lichen planus and (b) individuate an orodispersible dosage form suitable for its administration. IPECs were synthesized by mixing Eudragit^®^ E PO (EPO) and different grades of cross-linked polyacrylate derivatives, in different molar ratios, namely 1:1, 1:2, and 2:1. All IPECs resulted at nanoscale independently of their composition (120–200 nm). Both zeta-potentials (ζ) and mucoadhesive performances were influenced by the ratio between polymers. On the bases of the preliminary data, IPECs made of Polycarbophil and EPO in the 1:2 ratio were loaded with CP. The encapsulation efficiency was up 88% independently of the CP-IPEC ratio. The drug encapsulation caused IPEC destabilization in water, as it was noticed by the increase of ζ values and the formation of aggregates. Oral lyophilisates were prepared by freeze-drying slurries made of placebo or CP loaded IPECs, maltodextrin with a dextrose equivalent 38 and Span^®^80. The optimized formulation permitted to obtain a fast disintegration upon contact with water reducing the tendency of IPECs to aggregate. Moreover, oral lyophilisates allowed improving the apparent solubility of CP throughout the in vitro release experiment.

## 1. Introduction

Interpolyelectrolyte complexes (IPECs) are formed in aqueous dispersions by spontaneous association of oppositely charged polyelectrolytes due to strong but reversible electrostatic interactions [1]. The mild preparation procedure and responsiveness to various stimuli (i.e., pH, temperature, and osmolarity) without cross-linking agents or auxiliary molecules, e.g., catalysts, thereby reducing possible toxicity and other undesirable effects of the reagents. As the obtained polymeric networks are biocompatible and well-tolerated, they are exploited in drug delivery to administer both small drugs [2] and peptides or proteins by several routes, e.g., ocular [3], nasal [4], and oral [5].

Depending on the main features of selected polymers, IPECs exhibit peculiar physico-chemical properties due to their electrostatic interactions and flexibility. For instance, upon mixing two aqueous solutions of oppositely charged polyelectrolytes in a stoichiometric ratio, the resulting IPEC is insoluble and precipitates out [6], often as a colloid [7]. Then, the definition of a suitable drying technique, and the relative protocol, is required to improve their physical and microbiological stability. However, drying could also cause the formation of irreversible aggregates of irregular shape and size considering the IPEC dimensions.

**Scheme 1:**
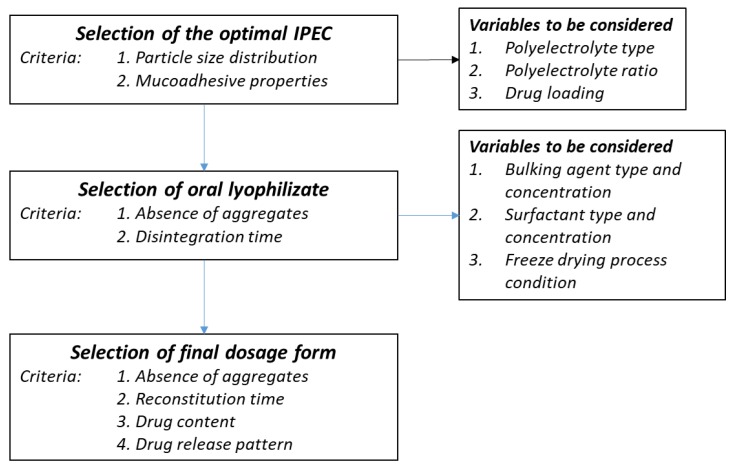
Technological steps of preparing oral lyophilisates containing IPECs.

Recently, a type of IPEC constituted by a poly(amino methacrylate) and an anionic polyacrylate derivative was proposed as mucoadhesive microparticles [8] which could be exploited in the treatment of buccal pathologies since it prolongs the residence time on a wide surface area. In contrast, the design of a suitable dosage form to administer a powder in the buccal cavity could be problematic in terms of dose accuracy and the easiness in handling. In the attempt to solve these issues, in this work we demonstrated the feasibility to prepare oral lyophilisates [9] containing mucoadhesive IPEC composed of Eudragit^®^ E PO (EPO) and Polycarbophil^®^. This material was chosen among a homogenous series of cross-linked polyacrylate derivatives able to provide the original suspension with unmodified particle size and size distribution, as detailed in Scheme 1. IPECs were loaded with Clobetasol proprionate (CP) selected as a model drug since it is mainstay of topical treatment for oral lichen planus (OLP) [10]. It should be noted that despite many international guidelines refer that its topical application allows good management of this condition reducing systemic side-effects [11], dosage forms intended for buccal route are still not available.

The experimental work was organized in three steps, as detailed in Scheme 1, which summarizesthe the selection criteria and the most important variables to be considered. Firstly, placebo IPECs made of EPO and four different types of carbomers were produced to elucidate the effect of the polycomplex composition on mucoadhesive properties and physico-chemical features. IPECs with satisfactory mucoadhesive properties were loaded with different amounts of CP to investigate the maximum loading ability of IPECs. Secondly, placebo and CP loaded IPECs were formulated as oral lyophisates using maltodextrin as main matrix forming materials due to its excellent water solubility [12]. Finally, considering the low aqueous solubility of CP (~4 mg/mL) [13], the possibility to improve the drug apparent solubility was also investigated.

## 2. Materials and Methods

### 2.1. Materials

Eudragit^®^ E PO (EPO)—a terpolymer of *N*,*N*-dimethylaminoethyl methacrylate (DMAEMA) with methylmethacrylate (MMA) and butylmethacrylate (BuMA), (PDMAEMA–*co*–MMA–*co*–BuMA) (mole ratio 2:1:1, MW 150 kDa) was used in this study as a cationic copolymer. Different grades of carbomer derivatives (Carbopol^®^ 71G NF polymer (C71G), Carbopol^®^ ETD 2020 NF polymer (C2020), Carbopol^®^ Ultrez 10 NF polymer (C10) and Noveon^®^ AA-1 Polycarbophil USP (NAA-1)) were used as polyanions. EPO and different types of Carbopol^®^ (C71G, C2020, C10) as well as Noveon^®^ AA-1 (NAA-1) were generously donated by Evonik Röhm GmbH (Darmstadt, Germany) and Lubrizol Advanced Materials (Wickliffe, OH, USA), respectively. Their main relevant physicochemical characteristics as specified by manufacturers are summarized in Table 1. The polymers were used after drying at 40 °C under vacuum over a 2 day-period. Maltodextrin with a dextrose equivalent 38 (Glucidex IT38, DS) was kindly gifted by Roquette (Lestrem, France). Span^®^80 and Tween^®^80 were provided by Croda Lubricants (Snaith, UK) and Carlo Erba Reagenti, (Milan, Italy), respectively. Clobetasol 17-propionate (CP) was purchased from SICOR (Pero, Italy). All solvents were of analytical grade, unless specified.

### 2.2. Synthesis of Placebo and CP Loaded IPECs

The conditions to optimize the interaction between chemically complementary grades of a polycationic (EPO) and a polyanionic (C71G, C2020, C10, NAA-1) polymer in the presence of CP were evaluated in an aqueous medium. EPO solution was obtained dissolving EPO in 1 M CH_3_COOH. Then, it was diluted with deionized water to the required volume and the pH was adjusted to 7.0 with 1 M NaOH. Carbomer dispersions were prepared by dispersing and swelling the polymer in 1 M NaOH. This dispersion was diluted with demineralized water to the desired volume and the pH was adjusted to 7.0 with 1 M CH_3_COOH. The EPO solutions were slowly poured into carbomer-CP dispersions [10]. The solutions and dispersions of copolymers and CP were mixed in different IPEC-CP weight ratios (e.g., 90:10, 80:20, 70:30, 60:40, 50:50 *w*/*w*), using three Carbomer/EPO ratios in synthesized IPECs (in equal quantities and with an excess of EPO or Carbopol^®^).

The optimal composition of IPEC (placebo) and IPEC-CP systems were obtained in a reactor system LR 1000 control equipped with pH- and temperature controlling units (IKA^®^, Staufen, Germany) under continuous agitation using overhead stirrer Eurostar 60 control (IKA^®^, Staufen, Germany) at 500 rpm. The feeding rate of EPO solution was about 2 mL/min and mixtures were stirred over a 7 day period. After the isolation, IPEC-CP particles were washed with ultrapure water (Smart2Pure UV/UF, Thermo Fisher Scientific, Waltham, MA, USA) and subsequently dried under vacuum at 40 °C (vacuum oven VD 23, Binder, Germany) over a 2 day period until constant weight. The samples were stored in tightly sealed containers at room temperature until use. The elementary analysis on placebo IPEC samples was carried out by a Thermo Flash 2000 CHNS/O elemental analyzer (Thermo Fisher Scientific, Paisley, UK).

### 2.3. IPEC Characterization

#### 2.3.1. Dynamic Light Scattering

To determine the hydrodynamic diameter (*D*_h_) of IPECs, laser diffraction analysis was carried out using a Zetasizer Nano ZS (Malvern Instruments, Worcestershire, UK). This technology determines particle sizes in the range from 0.5 nm to 5 μm allowing the detection of particle aggregates in a suspension. Since the particle stability was not sufficient in pure water during a single measurement, Span^®^80 at the concentration of 0.25% was used as a steric stabilizer. The analysis was conducted at a scattering angle of 173° and a temperature of 25 °C.

#### 2.3.2. Zeta-Potential Measurements

Charge was determined as the zeta potential (ζ) by using folded capillary cell at 25 °C using a Zetasizer Nano ZS (Malvern Instruments, Worcestershire, UK). The results are reported as mean ± standard deviation (*n* = 3).

#### 2.3.3. Modulated DSC Analysis

Thermal analysis on IPEC, CP, IPEC-CP were carried out using a modulated differential scanning calorimetry (MDSC; Discovery DSC™, TA Instruments, Newcaste, DE, USA), equipped with a refrigerated cooling system (RCS90, TA Instruments, Newcastle, DE, USA). Samples of about 5 mg exactly weighted were sealed in Tzero aluminium pans (TA Instruments, Newcastle, DE, USA) and empty pan was used as a reference. The mass of the reference and sample pans were considered to normalize the data. Dry nitrogen at a flow rate of 50 mL/min was used to purge the DSC cell. Indium and *n*-octadecane standards were used to calibrate the DSC temperature scale; enthalpic response and heat capacity were calibrated with indium and sapphire, respectively. The modulation parameters were set as follows: 2 °C/min heating rate, 40 s period and 1 °C amplitude. Samples were analyzed from 25 to 250 °C. Glass transition temperature was determined in the reversing heat flow signals by using TRIOS™ software (version 3.1.5.3696, TA Instruments, Newcastle, DE, USA).

#### 2.3.4. FTIR-Spectroscopy

ATR-FTIR-spectra were recorded using a Nicolet iS5 FTIR-spectrometer (Thermo Fisher Scientific, Waltham, MA, USA) equipped with a DTGS detector (Thermo Fisher Scientific, Waltham, MA, USA). IPECs, raw polymers (i.e., C71G, C2020, C10, NAA-1) and vacuum-dried IPEC-CP were directly placed on the iD5 smart single bounce ZnSe ATR crystal. The spectra were analysed using OMNIC spectra software (Thermo Fisher Scientific, Waltham, MA, USA).

### 2.4. In Vitro Mucoadhesive Properties of Placebo IPECs

The texture analysis was performed as previously described [14] using mucin as the adherent substrate [15]. Mucoadhesive properties were determined by using a software-controlled texture analyzer (Instron 5965, Instron, Pianezza, Italy) equipped with a 50 N force cell in adhesion mode. A flat faced compact of testing materials (weight: 170 mg, diameter: 11.28 mm) was obtained by applying a compression force of 10 tons for 30 s by means of a hydraulic press (Glenrothes, UK). Compacts were glued to the mobile steel punch. A mucin compact (weight: 130 mg, diameter: 11.28 mm) obtained applying a compression force of 10 tons for 60 s, was glued to a steel plate fixed at the bottom of the tensile apparatus. Both compacts were hydrated with 50 μL deionized water for 5 min to obtain a jelly layer. Upon making contact between the two hydrated compacts, a constant force of 1.3 N was applied over 360 s. The mucoadhesive properties were expressed as maximum detachment force (MDF), namely the force required to separate the IPEC compact from mucin upon an elongation of 25 mm at the rate of 0.1 mm/s; work of adhesion (WA), namely the area under the curve of the detachment force versus the elongation which represents the energy necessary to detach two compacts. Polyethylene plates and chitosan compacts were used as negative and positive control, respectively. The results are expressed as mean ± standard deviation (*n* = 4).

### 2.5. Preparation of Oral Lyophilisates

To set-up the freeze-drying parameters, the glass transition temperature of the maximally freeze-concentrated phase (*T*_g_′) of the aqueous solution of DS in the presence of different components was determined by a DSC 1 STARe System (Mettler Toledo, Greifensee, Switzerland). In brief, aliquots of about 30–40 mg were cooled below the expected *T*_g_′ at 1 K/min and kept at the temperature for 5 min. Thereafter, samples were re-heated at 5 K/min to room temperature. To optimize the tablet formulation, the effect of DS concentration and the presence of a surfactant (Span^®^80 or Tween^®^80) in different concentrations on the tablet disaggregation time and IPEC size were evaluated. The composition of tablets loaded with placebo IPECs is reported in Table 2.

Aliquots of 200 µL were poured into the cavity of PVC/OPA/Al/OPA/PVC laminate blister (Catalent Pharma Solutions, Somerset, NJ, USA) and loaded into an Epsilon 2–6 laboratory scale freeze-dryer (Martin Christ Freeze Dryers, Osterode, Germany). The samples were frozen at the rate of 1 K/min to a minimum shelf temperature of −25 °C including two equilibration steps at 5 and −5 °C for 15 min to achieve similar nucleation temperatures. After holding samples at −25 °C for 1 h, the chamber pressure was decreased to 0.120 mbar and the shelf temperature was increased to −10 °C at 1 K/min to initiate the main drying. After 6 h of sublimation, the shelf temperature was further increased to 40 °C at the rate of 1 K/min to initiate the secondary drying. The sublimation phase was carried out over a 5 h period. Then, samples sealed under vacuum in glass vials were stored at room temperature.

Tablets containing CP loaded IPECs were similarly prepared by weighing the exact amounts of IPEC containing 120 μg drug per single unit.

The oral lyophilisates were characterized in terms of uniformity of mass and disintegration time according to the Ph. Eur. 9th edition. After disintegrating one or two tablets in 10 mL of filtered deionized water (Milli-Q™ Water system, Millipore Corporation, Vimodrone, Italy), particle size and zeta potential of placebo and loaded IPECs were also measured.

### 2.6. In Vitro Drug Release Test

The in vitro drug release test was performed according to a “sample-and-separate” method [10]. Considering the limited volumes of fluids in the buccal cavity, the in vitro release test was carried out in oversaturation condition in order to better discriminate the different features of CP loaded IPECs. Oral lyophilisates were placed in closed glass vials containing 20 mL deionized water and shaken in a horizontal incubator at 50 strokes/min and 37 ± 0.5 °C. At each time point, a volume of 4 mL medium was diluted with 1 mL acetonitrile, and the amount of CP released was quantified by the high-performance liquid chromatography (HPLC) method reported in Section 2.7. The withdrawn medium was replaced with equal volumes of deionized water.

### 2.7. HPLC Method

The CP content loaded into IPECs and released in the in vitro release test was assayed by an HPLC method using HP1100 Chemstation (Agilent Technologies, Cernusco sul Naviglio, Italy). Chromatographic conditions: column: Spherisorb ODS2, 4.6 mm × 150 mm, 3 μm (Waters, Vimodrone, Italy); mobile phase: 50:50 (%*v/v*) acetonitrile:water; flow rate: 1.0 mL/min; wavelength: 240 nm; injection volume: 20 μL. The drug concentrations were determined from standard curves ranging from 0.1 to 50 μg/mL [9].

## 3. Results

### 3.1. Characterization of Placebo IPECs

**Figure 1:**
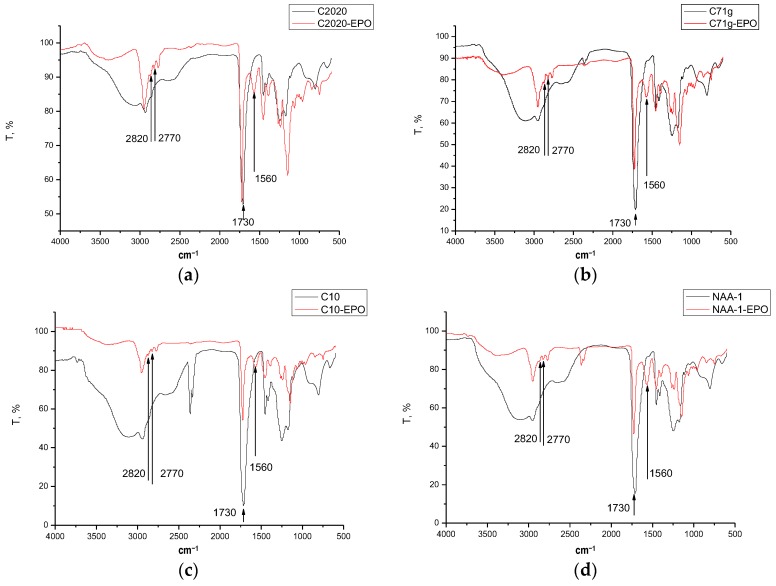
ATR-FTIR-spectra of C2020 and IPEC C2020/EPO (**a**); C71G and IPEC C71G/EPO (**b**); C10 and IPEC C10/EPO (**c**); and NAA-1 and IPEC NAA-1/EPO (**d**).

All IPECs obtained by mixing EPO and four types of carbomers differing in chemical composition, molecular weight, or cross-linking were insoluble in water. To evaluate the possible interactions between components, a physicochemical study was carried out by MDSC and FTIR spectroscopy. According to the FTIR spectra, a new absorption band at 1560 cm^−1^ appeared in the IPEC with respect to the raw materials, suggesting the formation of a new chemically individual compound (Figure 1).

This absorption band is diagnostic of the formation of ionic bonds between carboxyl groups of Carbopol^®^ and dimethylamino groups of EPO [16,17,18,19] and responsible for complex insolubilization. MDSC data supported the formation of such interaction at a molecular level. EPO and carbomer are both amorphous polymers with a characteristic *T*_g_ value (Table 3). After IPEC formation, a single value of *T*_g_ was detected independently of the nature of anionic polyelectrolytes suggesting the absence of microdomains of free copolymer. Moreover, the shift of *T*_g_ towards higher values with respect to the starting polymers, suggests the formation of a stiffer material. The elementary analysis of IPEC after washing revealed the presence of an excess of carbomer polymers in all samples (Table 3).

Regarding the particle size and particle size distribution, C10 and C2020, which provide substantially more viscous solutions than the low molecular weight counterpart, allowed the formation of nanoparticles (Table 4) and aggregates sizing about 5 μm. The percentage of this population of large particles considerably increased decreasing the EPO content (Table 4). In contrast, the mixing of NAA-1 and EPO led to the formation of nanoparticles of about 160 nm with a monomodal distribution (Table 4).

The evolution of zeta potential as a function of IPEC composition, which can be considered as an indication of the degree of inter-particle interaction, is summarized in Table 4. As the carbomer concentration increased from 33% to 67%, the zeta potential values shifted from 15 mV to a negative value according to the extent and the type of anionic copolymer used for the preparation of IPEC. This feature can be attributed to the presence of negatively charged carboxyl groups of Carbopol^®^, which do not participate to the formation of ionic bonds with the positively charged dimethylamino groups of EPO. Moreover, increasing the concentration of polyacrylate within the complex, the number of such carboxylic groups also increases, which give a negative charge to the IPEC particles.

The mucoadhesive properties (in terms of both WA and MDF) of the complexes made with EPO and different types of polyacrylate are reported in Table 4. All the IPEC compositions showed good mucoadhesive properties, since both MDF and WA were statistically higher than those measured using the negative control. As expected, the mucoadhesive performances of IPECs were influenced by the ratio between the polymers. The higher the EPO amount in each IPEC series, the higher the mucoadhesive properties. Indeed, when EPO concentration was 67%, the change of the IPEC charge from negative to positive values, as estimated by the zeta potential, allows the dimethylamino groups of EPO to interact by ionic bonds with the negatively charged ionized groups of sialic acid at the terminus of mucin subunits. The lower values of MDF, associated to a more negative of zeta potential, might be the result of greater repulsion between negative charges of mucin and IPECs.

These results indicate that mucoadhesion of IPECs made of C10, C71G, and NAA-1 are mainly attributed to the formation of electrostatic interactions. On the other hand, such mechanism of interaction cannot help to explain the behaviour of IPEC made with C2020, for which a less negative zeta potential in comparison with the raw polymer (C2020) was related to weaker mucoadhesion. Indeed, beyond the zeta potential values, other features of polymers (e.g., chemical composition and structure) can influence their ability to adhere to mucosa [20]. In the case of raw polymers, the more negative zeta potential may establish a better uncoiling to interpenetrate with oligosaccharide mucin chains. The mucoadhesion of C2020/EPO IPEC can be attributed by the formation of hydrogen bonds between carboxyl groups of Carbopol^®^ and mucin, since mucoadhesion of polymers containing weak anionic carboxyl, such as polyacrylic acid (Carbopol^®^), is often related to the formation of hydrogen bonds with mucin [21].

### 3.2. Oral Lyophilisates Containing Placebo IPEC

The physico-chemical characterization of placebo IPECs permitted to consider two materials worth of further characterization. In particular, C71G/EPO (50:50) and NAA-1/EPO (33:67) were chosen based on the zeta potential value (Table 4) to evaluate how the superficial properties can affect the formulation of oral lyophilisates and resuspendibility. In addition, IPEC made of NAA-1/EPO (33:67) is characterized by the highest rates of mucoadhesion (Table 4), which can also influence the properties of the final dosage form. To obtain oral lyophilisates with suitable characteristics, it is necessary to tune up both the formulation and the lyophilization parameters. Of fundamental importance to preserve IPEC nano-size during the lyophilization process is the selection of the type and concentration of lyoprotectants and steric stabilizers. The “vitrification hypothesis” suggests the possible role of lyoprotectants during freezing: saccharides form a glassy system, known also as cryo-concentrated phase, where nanoparticles are immobilized and preserved from the ice crystals [22]. Besides lyoprotectants, steric stabilizers can improve the nanoparticles stability during the lyophilization according to the “water replacement theory”. This theory suggests that the hydrogen bonds between water and nanoparticles are replaced by interactions occurring onto nanoparticles surface with the adsorbed steric stabilizer, thus avoiding particle aggregation or fusion [23]. Steric stabilizers are generally polymers and surfactants, such as polysorbates and poly(vinyl alcohol).

**Figure 2:**
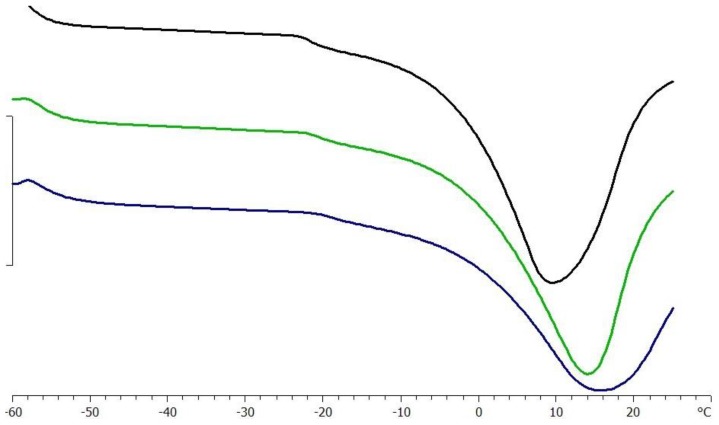
DSC analysis of 40% solution of DS 38 (black line) in the presence of 0.5% Span^®^80 (green line) and 10% IPEC No.1 (blue line).

Once both lyoprotectants and steric stabilizers are defined, an adequate lyophilization cycle is designed based on the *T*_g_′ and the *T*c. Indeed, the formulation is required to be cooled below its *T*_g_′ to assure the complete solidification [24] and *T*c, which is the maximum allowable temperature of product during primary drying, to avoid the collapse [25]. Thermal analysis indicated that the *T*_g_′ of a DS solution at 40% was −21.81 ± 0.3 °C without being significantly affected by the presence of the surfactant (*T*_g_′ = −20.56 ± 0.25 °C); meanwhile the dispersion of 10% IPEC caused a slight increase in *T*_g_′ value to −19.10 ± 0.04 °C, as exemplified in Figure 2. Hence, the samples were frozen at the temperature of −25 °C, considering a safety product margin of about 2 °C [21].

The final concentrations of the additives (i.e., lyoprotectants and steric stabilizers) in the formulations containing IPEC are reported in Table 2. All freeze-dried tablets loaded with IPEC presented as a white spongy texture. The tablets occupied the same volume of the original frozen mass and no shrinkage or cake collapse was observed, demonstrating that the process parameters yielded good lyophilisates.

Tablets obtained by the DS solution at the highest concentration presented a very irregular surface due to the presence of bubble after filling the blisters. Decreasing the DS concentration to about 40%, visually acceptable tablets were obtained and, therefore, they were disintegrated in water in order to characterize IPECs in terms of particle size and PDI. The presence of a steric stabilizer was essential during lyophilization since DS as such was not able to avoid the formation of large and irreversible aggregates (Table 2). However, sticky tablets, difficult to handle, were obtained by using Tween^®^80 independently of its concentration and, therefore, discarded from further evaluation. Span^®^80 was effective as a steric stabilizer as a function of its concentration since only the formulation containing DS in combination with 0.5% of Span^®^80 preserved the IPEC size upon lyophilization (Table 2). Additionally, the ratio between the cryoprotectant and IPEC influenced the freeze-drying process, since at 20% IPEC loading the resuspended particles exhibited a monomodal distribution with a low size heterogeneity (PDI ~ 0.15). This evidence agreed with the results on the lyopresevation effect of threalose on diblock and triblock poly(lactic acid)-poly(ethylene oxide) copolymer nanoparticles—the lyoprotective efficiency increased at higher nanoparticles concentration [26].

After lyophilization and redispersion, IPECs shifted their characteristic surface charge from about 15 to −25 mV (Table 2). This variation can be the result of a “masking-effect” due to the adsorption of maltodextrin on the positive surface of IPEC. This result is in line with literature data since the entrapment of nanoparticles in some polymers usually modifies the zeta potential because the coating layers shield the surface charge and move the shear plane out wards from the particle surface [27,28,29].

According to the obtained results, the optimal composition was 20%, IPEC NAA-1/EPO (33:67), 40.9% DS, and 0.5% Span^®^80 since the resulting oral lyophilisates have the required disintegration time (<30 s) and after disintegration test IPEC particles had a monomodal distribution without aggregates. Thus, this composition was selected to produce oral lyophilisates loaded with CP.

### 3.3. CP Loaded IPECs

The effect of different CP loading on the main features of IPEC made of NAA-1 and EPO in the ratio 33:67 was evaluated. The loading procedure gave a high encapsulation efficiency in all considered ratios (Table 5).

**Figure 3:**
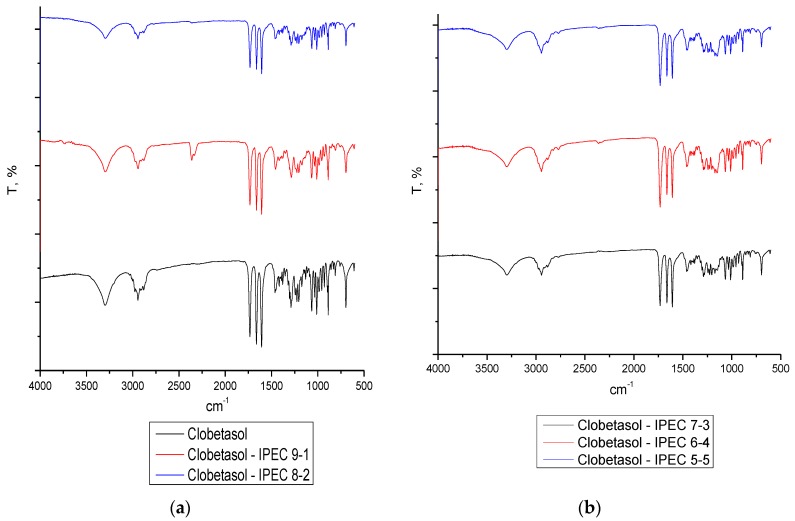
ATR-FTIR-spectra of raw material CP (panel (**a**)) and CP loaded IPECs in the following ratio: (panel (**a**)) 9:1, 8:2; (panel (**b**)) 7:3, 6:4, 5:5.

The FTIR spectra of CP loaded IPECs revealed that no interactions occurred independently on their ratio (Figure 3).

**Figure 4:**
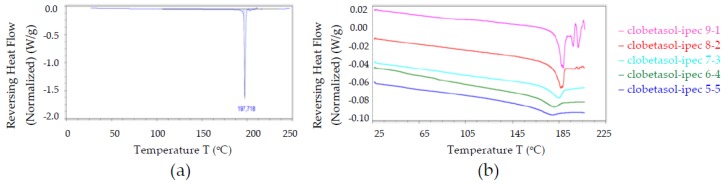
MDSC thermograms of (**a**) CP raw material and (**b**) CP loaded CP—in different ratios, namely 90:10, 80:20, 70:30, 60:40, and 50:50.

This result is consistent with the MDSC data as the endothermic event attributed to CP melting was observed in all samples and it increased the drug content accordingly (Figure 4). Independently of the drug content, IPEC dimension increased from around 160 to 450 nm (Table 5). This behavior can be due to the presence of aggregates since the drug loading caused a shift of zeta potential value in the range of instability (Table 5).

As far as the mucoadhesion is concerned, the drug loading led to a decrease in the MDF values of IPECs (Table 5), at a higher extent with increasing CP content. As a matter of fact, the values obtained for CP content higher than 70% (i.e., formulations CP-IPEC 80:20 and 90:10) resulted not significantly different from the negative control. On the other hand, for all the tested formulations, WA was higher than the negative control and, for those with low CP content (i.e., formulations CP-IPEC 50:50, 60:40, and 70:30), it was of the same order of magnitude of the corresponding placebo IPECs.

In the case of drug loaded IPECs, the decrease in MDF, not concurrent with a decrease in WA, could be due to an increase of the viscous modulus of the hydrated interpolymeric complex/mucin mixture. As a matter of fact, the last phase during the separation of the drug loaded IPECs showed a resistance to the detachment in terms of elongation and time, higher than the placebo. Indeed, the mucin compact was not totally detached from IPEC compact due to the formation of visually observed fibrils. Oral lyophilisates were obtained by dispersing an appropriate amount of CP loaded IPECs in the DS solution in order to have a drug content of 120 μg/unit. The final composition is reported in Table 6. After freeze-drying all tablets appeared as elegant solids without defects or sign of collapse, easy to remove from blister and to handle. The disintegration time of all oral lyophilisates was less than 30 s and no aggregates were detected confirming suitability of components to stabilize IPECs during the lyophilization process (Table 6). The zeta potential values of all resuspended IPECs shifted towards the neutrality probably because the excipients remained adsorbed on the IPEC surface.

**Figure 5:**
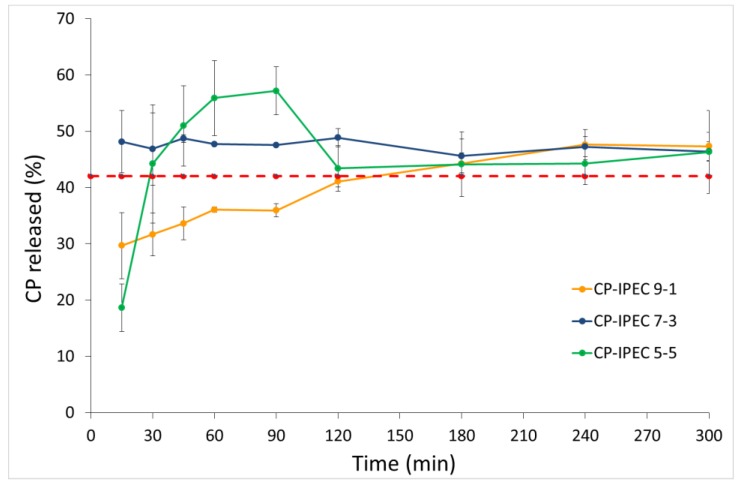
In vitro dissolution profile of CP from oral lyophilisates containing CP-IPEC *w/w*: 9:1 (90:10), 7:3 (70:30), and 5:5 (50:50) under oversaturation conditions. The dotted line corresponds to the CP solubility at the saturation.

The dissolution profiles showed that the CP encapsulation into IPEC improved its apparent solubility as a function of the loaded drug amount (Figure 5). Indeed, the CP-IPEC ratio of 50:50 exhibited the highest supersaturation degree, which was conversely unstable, since after 120 min the concentration of CP in the dissolution medium was superimposable to that of CP solubility. On the other hand, at the CP-IPEC ratio of 90:10 (Formulation 5) the steady state was reached in about 2 h. Based on these observations, it can be assumed that IPEC not only controlled the drug release rate, but also favored the stabilization of the supersaturated system. Indeed, when the CP amount ranged from 80% to 60% (Formulations 2–4, Table 6), a stable supersatured solution was obtained over the entire considered period of time.

## 4. Conclusions

A new drug delivery system obtained combining the colloidal and mucoadhesive properties of IPEC formed by Carbopol^®^ and EPO, was proposed to treat buccal pathologies. In particular, the ability to interact with mucin was attributed to the IPEC structural features, namely the presence of free dimethylamino groups of EPO or carboxylate groups of Carbopol^®^. Indeed, the higher the mucoadhesion, the higher the excess of unbalanced charge in IPEC. Regarding oral lyophilisates, the use of maltodextrin DE 38 and Span^®^80 preserved the IPEC size during the thermal stress so that it was possible to reconstitute the original nanosuspension upon contact with water in few seconds. Moreover, this approach allowed to improve the CP apparent solubility thanks to the formation of a stable supersaturated system.

Hence, the overall data suggest that this dosage form could be advantageously exploited in drug delivery systems as demonstrated in the case of clobetasol propionate.

The performed work also permitted us to withdraw general information on the design of oral lyophilisates loaded with nanosized particles. Scheme 1 detained the general approach, underling the selection criteria of each phase and the most important variables to be considered.

## Figures and Tables

**Table 1:** Physicochemical characteristics as specified by Evonik (healthcare.evonik.com) for Eudragit^®^ E PO (EPO) and Lubrizol Advanced Materials (lubrizol.com) for polycarbophil and different grades of carbomer used to prepare interpolyelectrolyte complex (IPEC). (Abbreviation: Monomers: AA—acrylic acid; EG—ethylene glycol; PAA—polyacrylic acid; crosslinking agents: AEPE—allyl ester of pentaerythritol; DVG—divinyl glycol).

Grade (Code)	Type of Polymer	Viscosity (0.5%, pH 7.5), cP	MW (kDa)	Mesh Size—Distance between Crosslinks (kDa)	ζ (mV)
C2020	Carbomer interpolymer. Type B (copolymer of AA and EG crosslinked with AEPE)	47,000–77,000	4500	11.4	−36.2 ± 2.7
C71G	Carbomer homopolymer. Type A (PAA, crosslinked with AEPE)	4000–11,000	3000	237.6	−51.0 ± 2.0
C10 *	Carbomer interpolymer. Type A (block copolymer of EG and a long chain alkyl acid ester)	45,000–65,000	3000	- *	−33.3 ± 2.1
NAA-1 *	Polycarbophil (PAA, crosslinked with DVG)	2000–12,000	3000	- *	−40.1 ± 2.1
EPO	Butylated methacrylate copolymer	-	150	-	45.7 ± 2.1

* No data available.

**Table 2:** Tablet composition and its influence on hydrodynamic diameter (*D*_h_) and zeta potential (ζ) of placebo IPECs after disintegration.

Tablet Code	Tablet Composition (%, *w/w*)					Disintegration Time (s)	*D*_h_ (nm)	ζ (mV)
	IPEC 1	IPEC 8	DS	S80	T80			
1	10%	-	40.9	-	-	6	149 ± 42 (54.9%)	n.d.
2	10%	-	81.8	-	-	n.d. *	-	-
3	-	10%	40.9	-	-	11	136 ± 16 (95.6%)	−26.6 ± 0.7
4	-	10%	81.8	-	-	n.d. *	-	-
5	10%	-	40.9	0.01	-	20	141 ± 0 (97.8%)	−26.6 ± 0.6
6	10%	-	40.9	0.1	-	6	132 ± 8 (90.0%)	−25.8 ± 0.1
7	10%	-	40.9	0.5	-	26	145 ± 8 (98.4%)	−26.0 ± 1.3
8	10%	-	40.9	-	0.01	n.d. **	-	-
9	10%	-	40.9	-	0.1	n.d. **	-	-
10	10%	-	40.9	-	0.5	n.d. **	-	-
11	-	10%	40.9	0.01	-	33	125 ± 10 (100%)	−26.3 ± 1.9
12	-	10%	40.9	0.1	-	12	111 ± 9 (100%)	−25.1 ± 3.0
13	-	10%	40.9	0.5	-	15	114 ± 12 (98.2%)	−19.0 ± 2.1
14	-	10%	40.9	-	0.01	n.d. **	-	-
15	-	10%	40.9	-	0.1	n.d. **	-	-
16	-	10%	40.9	-	0.5	n.d. **	-	-
17	20%	-	40.9	0.5	-	26	165 ± 1 (100%)	−46.9 ± 1.2

* Tablets presented an irregular surface due to the presence of bubbles. ** Tablets were sticky and, therefore, difficult to handle.

**Table 3:** MDSC data on raw materials and placebo IPECs.

Samples Name	*T*_g_ (°C)	Composition (mol/mol)
EPO	52.1 ± 1.2	-
C71G	129.5 ± 1.2	-
C2020	125.2 ± 1.2	-
C10	126.5 ± 1.2	-
NAA-1	131.3 ± 1.5	-
C71G/EPO	132.8 ± 1.2	2.12:1 *
C2020/EPO	133.5 ± 1.2	1.73:1 *
C10/EPO	134.8 ± 1.1	2.11:1 *
NAA-1/EPO	138.5 ± 1.3	1.66:1 *

* The composition of carbomer/EPO (mol/mol) IPECs is referred to the elementary analysis.

**Table 4:** Main physical features, namely hydrodynamic diameter (*D*_h_), zeta potential (ζ), and mucoadhesive properties of placebo IPECs. The value in the brackets refers to the percentage of the main population.

IPEC Code	Composition (%, *w/w*)					ζ (mV)	*D*_h_ (nm)	MDF (kPa)	WA (mJ)
	EPO	NAA1	C10	C71G	C2020				
1	67	33	-	-	-	14.7 ± 0.5	154 ± 9 (98.0%)	111 ± 10	2611 ± 413
2	50	50	-	-	-	−5.5 ± 2.5	154 ± 0 (97.6%)	100 ± 13	1848 ± 335
3	33	67	-	-	-	−16.0 ± 0.4	168 ± 4 (96.1%)	49 ± 6	741 ± 33
4	67	-	33	-	-	7.7 ± 1.0	222 ± 9 (94.1%)	85 ± 20	1462 ± 210
5	50	-	50	-	-	−5.1 ± 0.9	158 ± 4 (96.2%)	103 ± 1	2154 ± 66
6	33	-	67	-	-	−13.7 ± 0.5	149 ± 13 (52.7%)	45 ± 6	643 ± 57
7	67	-	-	33	-	4.3 ± 1.6	195 ± 12 (63.7%)	94 ± 17	1902 ± 322
8	50	-	-	50	-	−47.8 ± 2.7	175 ± 17 (97.2%)	67 ± 31	1272 ± 448
9	33	-	-	67	-	−28.8 ± 1.7	188 ± 9 (96.4%)	55 ± 9	1010 ± 95
10	67	-	-	-	33	−28.2 ± 4.0	166 ± 9 (90.0%)	89 ± 25	656 ± 51
11	50	-	-	-	50	−28.2 ± 3.5	192 ± 11 (83.3%)	43 ± 2	678 ± 25
12	33	-	-	-	67	−20.6 ± 1.8	225 ± 9 (71.9%)	57 ± 5	802 ± 109

Chitosan (positive control): MDF = 62 ± 10 kPa; WA = 1682 ± 162 mJ. Polyethylene (negative control): MDF = 13 ± 1 kPa; WA = 407 ± 175 mJ.

**Table 5:** Characterization of clobetasol propionate (CP) loaded into IPEC formed by NAA-1 and EPO in the ratio 33:67 %.

Composition	CP Content (%)		*D*_h_ (nm)	ζ (mV)	MDF (kPa)	WA (mJ)
	Theoretic	Actual				
CP-IPEC 50:50	50	53.2 ± 0.3	401 ± 295	−11.3 ± 2.1	30 ± 6	2530 ± 58
CP-IPEC 60:40	60	56.1 ± 1.4	435 ± 197	−9.0 ± 1.2	22 ± 5	2988 ± 110
CP-IPEC 70:30	70	62.1 ± 5.3	431 ± 119	−10.8 ± 1.2	18 ± 2	2550 ± 27
CP-IPEC 80:20	80	79.4 ± 0.2	560 ± 116	6.8 ± 2.8	16 ± 2	1400 ± 26
CP-IPEC 90:10	90	88.5 ± 12.6	416 ± 6	3.8 ± 2.2	15 ± 1	820 ± 19

**Table 6:** Composition of oral lyophilisates containing CP loaded IPECs formed by NAA-1 and EPO in the ratio 33:67 %. The amount of IPEC was defined in order to have 120 μg drug per unit. The main features of CP loaded IPECs after disintegration were evaluated in terms of particle size (*D*_h_) and zeta-potential (ζ).

Formulation	Tablet Composition (%*w/w*)				*D*_h_	Disintegration		
	Composition	IPEC	Span^®^80	DS		Time (s)	*D*_h_ (nm)	ζ (mV)
1	CP-IPEC 50:50	0.29	1.20	98.50	299 ± 122 (95.6%)	<30	323 ± 69	1.08 ± 0.16
2	CP-IPEC 60:40	0.24	1.21	98.55	419 ± 81	<30	363 ± 122	−0.99 ± 1.25
3	CP-IPEC 70:30	0.21	1.21	98.59	306 ± 198	<30	350 ± 87	−4.42 ± 0.46
4	CP-IPEC 80:20	0.18	1.21	98.61	369 ± 178	<30	448 ± 122	−2.90 ± 1.64
5	CP-IPEC 90:10	0.16	1.21	98.63	518 ± 88	<30	356 ± 70	3.27 ± 0.60
